# An Aging-Related Gene Signature-Based Model for Risk Stratification and Prognosis Prediction in Lung Squamous Carcinoma

**DOI:** 10.3389/fcell.2022.770550

**Published:** 2022-03-01

**Authors:** Wen-Yu Zhai, Fang-Fang Duan, Si Chen, Jun-Ye Wang, Ze-Rui Zhao, Yi-Zhi Wang, Bing-Yu Rao, Yao-Bin Lin, Hao Long

**Affiliations:** ^1^ State Key Laboratory of Oncology in Southern China, Collaborative Innovation Center for Cancer Medicine, Department of Thoracic Surgery, Sun Yat-Sen University Cancer Center, Guangzhou, China; ^2^ Lung Cancer Research Center, Sun Yat-Sen University, Guangzhou, China; ^3^ State Key Laboratory of Oncology in Southern China, Collaborative Innovation Center for Cancer Medicine, Department of Medical Oncology, Sun Yat-Sen University Cancer Center, Guangzhou, China

**Keywords:** lung squamous carcinoma (LUSC), aging, prognostic signature, risk stratification, anti-tumor immune cells infiltration

## Abstract

Aging is an inevitable process characterized by a decline in many physiological activities, and has been known as a significant risk factor for many kinds of malignancies, but there are few studies about aging-related genes (ARGs) in lung squamous carcinoma (LUSC). We designed this study to explore the prognostic value of ARGs and establish an ARG-based prognosis signature for LUSC patients. RNA-sequencing and corresponding clinicopathological data of patients with LUSC were downloaded from The Cancer Genome Atlas (TCGA) and Gene Expression Omnibus (GEO). The ARG risk signature was developed on the basis of results of LASSO and multivariate Cox analysis in the TCGA training dataset (*n* = 492). Furthermore, the GSE73403 dataset (*n* = 69) validated the prognostic performance of this ARG signature. Immunohistochemistry (IHC) staining was used to verify the expression of the ARGs in the signature. A five ARG-based signature, including A2M, CHEK2, ELN, FOS, and PLAU, was constructed in the TCGA dataset, and stratified patients into low- and high-risk groups with significantly different overall survival (OS) rates. The ARG risk score remained to be considered as an independent indicator of OS in the multivariate Cox regression model for LUSC patients. Then, a prognostic nomogram incorporating the ARG risk score with T-, N-, and M-classification was established. It achieved a good discriminative ability with a C-index of 0.628 (95% confidence interval [CI]: 0.586–0.671) in the TCGA cohort and 0.648 (95% CI: 0.535–0.762) in the GSE73403 dataset. Calibration curves displayed excellent agreement between the actual observations and the nomogram-predicted survival. The IHC staining discovered that these five ARGs were overexpression in LUSC tissues. Besides, the immune infiltration analysis in the TCGA cohort represented a distinctly differentiated infiltration of anti-tumor immune cells between the low- and high-risk groups. We identified a novel ARG-related prognostic signature, which may serve as a potential biomarker for individualized survival predictions and personalized therapeutic recommendation of anti-tumor immunity for patients with LUSC.

## Introduction

Lung cancer (LC), the second most commonly diagnosed malignancy annually, is the leading cause of tumor-related death worldwide (Sung et al., 2020). Lung squamous carcinoma (LUSC), one of the major histological types of LC (Santarpia et al., 2018), occupies about 25% to 30% of non-small cell lung cancer (NSCLC) (Travis et al., 2015). The diagnosis and treatment of LC, especially targeted therapy, have substantially improved during the last decades. Unlike patients with lung adenocarcinoma (LUAD), only few patients with LUSC benefit due to the different gene mutation profiles (Yu et al., 2016; Oberndorfer and Müllauer, 2018). And the improvement of overall survival (OS) in LUSC patients remains dissatisfactory (Piperdi et al., 2014; Siegel et al., 2019). Until now, the tumor-node-metastasis (TNM) system has been commonly adopted to predict individual clinical outcomes, but it contains limited factors and neglects genetic characteristics (Balachandran et al., 2015). Thus, it is vital to develop individual antineoplastic protocols and exploit new prognostic biomarkers for identifying heterogeneous patients with LUSC and guiding personalized therapeutic care.

Aging, which is characterized by gradual functional deterioration of many tissues, is an inevitable and important biological process overtime, lastly generating numerous chronic and age-related pathologies, and is a powerful risk factor for several kinds of diseases, including neoplastic, neurodegenerative, metabolic, and cardiovascular diseases (havlakadze, 2019; Armanios et al., 2015; Benayoun et al., 2019; Smetana et al., 2016). Cytologically speaking, aging is correlated with mitochondrial dysfunction, genomic instability, cellular senescence, and so on, which is accompanied by the accumulation of irreparable damage and lethal substances in cells (Yin and Chen, 2005; López-Otín et al., 2013). Aging has been found to have an effect of irreversibly arresting cell growth and development, inhibiting the uncontrolled proliferation of tumor cells (Mosteiro et al., 2016; He and Sharpless, 2017; Calcinotto et al., 2019). A large flat morphology and reduced motility in senescent cells may contribute to suppress invasion, escape, cell migration, and metastasis (Zhao et al., 2015). However, the mechanisms and impact of cell aging on malignant tumors are quite complicated. Aging-related genes (ARGs) play a vital role in initiation and regulation of cell aging, and potentially affect tumor cells in complex ways. Regulation of tumor cellular senescence by ARGs can inhibit tumors, but ARGs can potentially promote tumor initiation, development, and metastasis (Johnson et al., 2013; Mosteiro et al., 2016; Galluzzi et al., 2018; Calcinotto et al., 2019; Lee and Schmitt, 2019). Lately, the potential diagnostic or prognostic value of ARGs have been explored and confirmed in colorectal cancer and LUAD (Xu and Chen, 2021; Yue et al., 2021). But its prognostic values and potential mechanisms in LUSC remain unknown, and no precise ARG-based risk signature has been developed for LUSC patients.

A comprehensive model with multi-genes shows stronger predictive capacity than a model with one gene (Srivastava and Gopal-Srivastava, 2002). Thus, in the current study, we made use of The Cancer Genome Atlas (TCGA) database to establish an ARG-based signature to predict individual prognosis for LUSC, and the data of a Gene Expression Omnibus (GEO) dataset validated the prognostic value of the ARG-based signature. Finally, a predictive nomogram including the ARG-based signature and TNM system was developed for precise survival predictions of LUAC.

## Materials and Methods

### Data Collection and Preparation

Data of gene expression and clinical information of LUSC patients were downloaded from the TCGA (https://tcga-data.nci.nih.gov/tcga/) and GEO databases (https://www.ncbi.nlm. nih. gov/geo/). After excluding cases with incomplete clinical information and follow-up of less than 1 day, 492 patients from the TCGA dataset were analyzed as a training set and 69 patients from the GSE73403 dataset were used for validation. The scale method from the ‘limma’ R package was performed to normalize gene expression profiles. The Masked Somatic Mutation data (varscan. Somatic. Maf) were downloaded and analyzed by the “maftools” R package (Mayakonda and Koeffler, 2016). A total of 309 human ARGs were obtained from the Human Aging Genomic Resources 3 (Supplementary Table S1).

### Construction and Validation of the Prognostic ARG Signature

A log2 | fold change | > 2 and false discovery rate (FDR) < 0.05 were defined as the cut-off values. The differentially expressed genes (DEGs) in LUSC tumor tissues and normal tissues were analyzed by the R software “limma” package. The ARG candidates for the prognostic-related signature were firstly selected using univariate Cox regression analysis (*p* < 0.05) in the TCGA cohort. Secondly, we used the least absolute shrinkage and selection operator (LASSO) regression model to further narrow down the number of prognostic-related ARG candidates. Then, the multivariate Cox model was used to assess the prognostic contributions of each ARG candidate in OS and determine the best weighting coefficient of each prognostic-related ARG candidate. Finally, the signature enrolled all the differentially expressed prognostic-related ARGs. The risk score of each patient was summed up by normalized expression levels of ARGs and their corresponding regression coefficients. The specific formula was as follows: Risk score = sum (each ARG’s expression level × corresponding coefficients). According to the cut-off point of risk scores derived from maximally selected log-rank statistics, LUSC patients in the TCGA training cohort were divided into low- and high-risk groups. The Kaplan-Meier method was utilized to estimate OS and the log-rank test was used to compare the differences of OS between the two groups.

To validate this prognostic signature, the risk score of patients in the GSE73043 dataset was calculated according to the same formula as the TCGA cohort. Patients in the GSE73043 validation cohort were also divided into two groups according to the cut-off points of risk scores from maximally selected log-rank statistics. Kaplan-Meier curves and the log-rank test were performed to identified the relation between the ARG signature and OS in the validation cohort.

### Gene Set Enrichment Analyses

To explore the potential molecular mechanisms of the ARGs, we performed Gene Set Enrichment Analysis (GSEA) to find enriched terms between high- and low-risk patients (Subramanian et al., 2005). GSEA was performed in Java GSEA v. 4.0.1 with Kyoto Encyclopedia of Genes and Genomes (KEGG) pathways in C2 and Gene Ontology (GO) terms in C5. After performing 1,000 permutations, genes with a false discovery rate q < 0.05 were seen as significantly enriched.

### Immune Infiltration and Tumor Mutation Burden Analyses

After normalizing the expression data in the TCGA dataset, we used single-sample GSEA (ssGSEA) to evaluate 28 immune cells in the R package “GSVA” (Tamborero, et al., 2018) with 782 genes included in the gene sets (http://software.broadinstitute.org/gsea/msigdb/index.jsp). With the perm set to 1,000, the CIBERSORT algorithm was used in the CIBERSORT software package to evaluate the proportion of 22 types of infiltrating immune cells based on LM22 (Newman et al., 2015). Immune scores and stromal scores in low- and high-risk groups were calculated by the R package ‘ESTIMATE’. According to the length of the human exon, the TMB calculated for each patient was equal to the total mutation frequency/35 MB. Dividing the total number of mutations by the size of the coding region of the target results in TMB per megabase. The Wilcoxon test and Mann-Whitney *U* test were performed to contrast the differential abundances of immune infiltrates, expression level of PD-1, PD-L1, PD-L2, and CTLA4, TMB, immune score, and stromal score between the low- and high-risk groups.

### Establishment and Validation of a Predictive Nomogram

In the TCGA dataset, we established a nomogram integrating the ARG signature and TNM staging system for predicting individual survival. Besides, calibration curves for 1-, 3-, and 5 years OS were calculated to evaluate the predictive accuracy of our nomogram in the TCGA dataset as well as the GSE73403 validation dataset.

### Immunohistochemistry

Tumor and adjacent non-tumorous tissue specimens from 10 LUSC samples from Sun Yat-Sen University Cancer Center between January 2016 and January 2017 were collected for the immunohistochemistry (IHC) assay. The formalin-fixed, paraffin-embedded tissue sections were dewaxed and rehydrated followed by antigen retrieval and blocking.

The slides were incubated with primary antibodies, rabbit anti-CHEK2 polyclonal antibody (1:2000; ab207446; ABCAM), rabbit anti-A2M polyclonal antibody (1:500; ab109422; ABCAM), rabbit anti-ELN polyclonal antibody (1:2000; ab213720; ABCAM), rabbit anti-c-FOS polyclonal antibody (1:2000; ab214672; ABCAM), and rabbit anti-PLAU polyclonal antibody (1:150; ab133563; ABCAM) overnight in 4°C.

After being incubated with anti-rabbit secondary antibody, HRP-conjugated rabbit polymer (1:500; ab97051; ABCAM) and liquid diaminobenzidine tetrahydrochloride plus substrate (DAB chromogen, Changjia) were used for visualization followed by counterstaining with hematoxylin. The samples were photographed by microscope (Nikon), and images were analyzed using ImageJ FIJI v2.1.0.

Ten random fields were analyzed per tissue section for semi-quantitative scoring, and the scoring method was as follows: 1) Positive cell rate score 0 for <10% positive cells, one for 10∼25% positive cells, two for 25∼50% positive cells, three for 50∼75% positive cells, and four for >75% positive cells.

We also used the Human Protein Atlas database to identify the protein expression of immunohistochemical staining of these five ARGs in LUSC patients.

### Statistical Analysis

Continuous data were shown as the mean ± SD and compared by Student’s *t*-test. Categorical variables were listed as frequencies with percentages and tested using the chi-square (*χ* ^2^) test. According to an outcome-oriented approach for OS, maximally selected log-rank statistics from the “maxstat” R package were collated to decide the optimal cut-off value of risk scores for risk stratification (Hothorn and Zeileis, 2008). Survival curves were estimated by the Kaplan-Meier method, and differences between these two different risk groups were compared with the log-rank test. Based on results from the multivariate Cox model, we established a predictive nomogram using the “rms” R package, and assessed its predictive accuracy by calibration curves and compared its discriminate ability using time-dependent receiver operating characteristic (ROC) curves. Statistical analysis was conducted using SPSS (version 22.0) and R software (version 4.0.1); a *p* value <0.05 was considered as statistical significant.

## Results

### Identifying a Prognosis-Related ARG Signature

**FIGURE 1:**
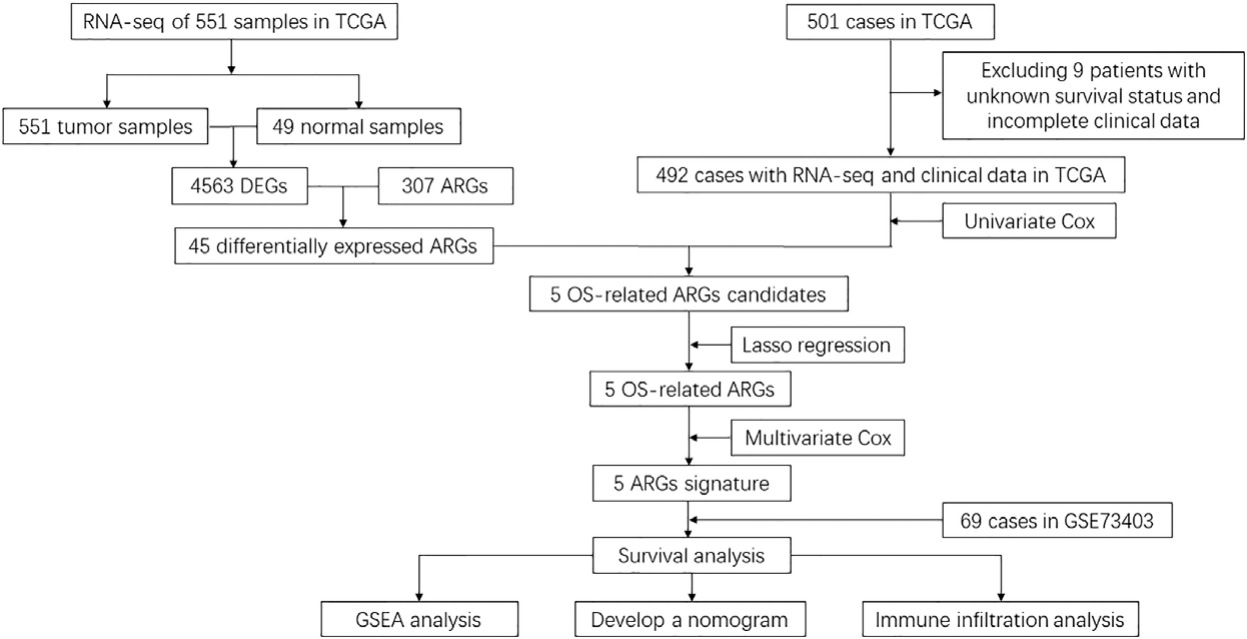
Flowchart of data collection and analysis.

As represented in the flowchart (Figure 1), gene differential expression analysis was performed in the TCGA dataset between 502 tumor tissues and 49 normal tissues. We discovered 1924 upregulated and 2,639 downregulated DEGs including 45 differentially expressed ARGs. After excluding 9 cases lacking survival or important clinical data, 492 cases from the TCGA training cohort were included in this study to decide prognosis-related ARGs as well as construct the ARG-based signature. In addition, 69 cases from the GSE73403 validation cohort were used to validate the ARG-based signature. Clinicopathological factors of the TCGA training cohort and the GSE73403 cohort are shown in Table 1.

**TABLE 1:** Patients’ characteristics.

	TCGA training cohort *n* = 492	GEO validation cohort *n* = 69
Gender		
Male	364 (74.0)	65 (94.2)
Female	128 (26.0)	4 (5.8)
Age (year)	61.3 ± 9.6	58.3 ± 8.5
≤65	171 (34.8)	44 (63.8)
>65	321 (65.2)	25 (36.2)
Smoking history		
Yes or ever or unknown	474 (96.3)	58 (84.1)
No	18 (3.7)	11 (15.9)
T stage		
T1	114 (23.2)	4 (5.8)
T2	286 (58.1)	42 (60.9)
T3	69 (14.0)	20 (29.0)
T4	23 (4.7)	3 (4.3)
N stage		
N0	320 (65.0)	35 (50.7)
N1	127 (25.8)	17 (24.6)
N2	40 (8.1)	17 (24.6)
N3	5 (1.0)	0 (0)
M stage		
M0	486 (98.8)	69 (100)
M1	6 (1.2)	0 (0)

**FIGURE 2:**
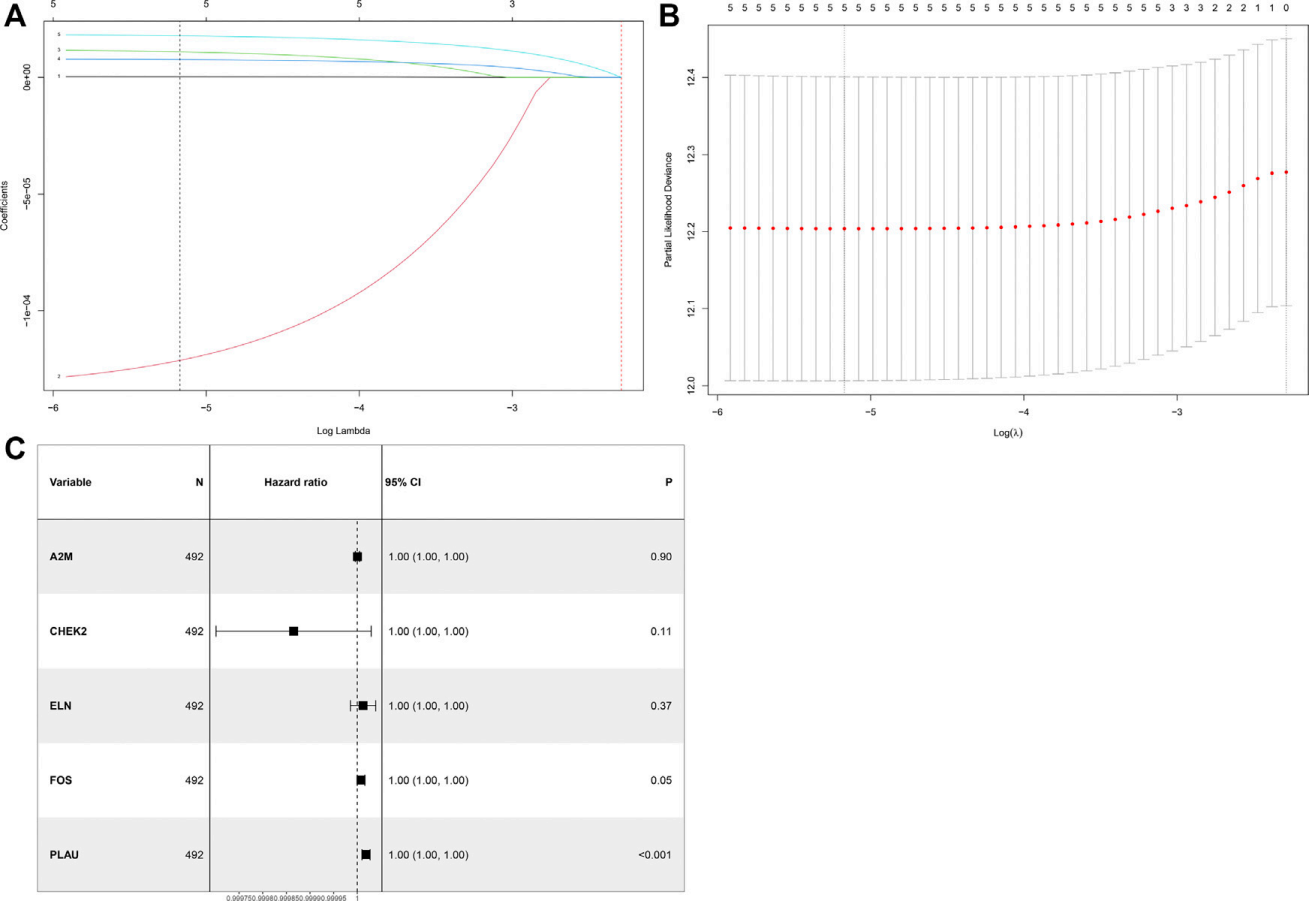
Identification of a prognosis-related ARG-based signature in the TCGA training cohort. **(A)** Selection of the optimal candidate genes in the LASSO model. **(B)** LASSO coefficients of prognosis-associated ARGs, each curve represents a gene. **(C)** Forest plots showing results of univariate Cox regression analysis between the candidate ARG expression and overall survival.

After univariate Cox analysis by using mRNA expression profiles of each differentially expressed ARG, five OS-related ARGs from TCGA were identified (Supplementary Table S2), which were also significant in the LASSO Cox regression analysis (Figures 2A,B) and entered the multivariate Cox regression analysis (Figure 2C). Finally, a five-ARG risk signature was constructed according to 492 LUSC cases in the TCGA dataset, whose risk score was specifically calculated based on a linear combination of gene expression levels and their corresponding regression coefficients from the multivariate Cox analysis. The specific formula was as follows: Risk score = *A2M* × 2.952e-7 - *CHEK2* × 1.347e-4 + *ELN* × 1.222e-5 + *FOS* × 7.953e-6 + *PLAU* × 1.858e-5.

### Prognostic Value of the ARG Signature in the Training Cohort

**FIGURE 3:**
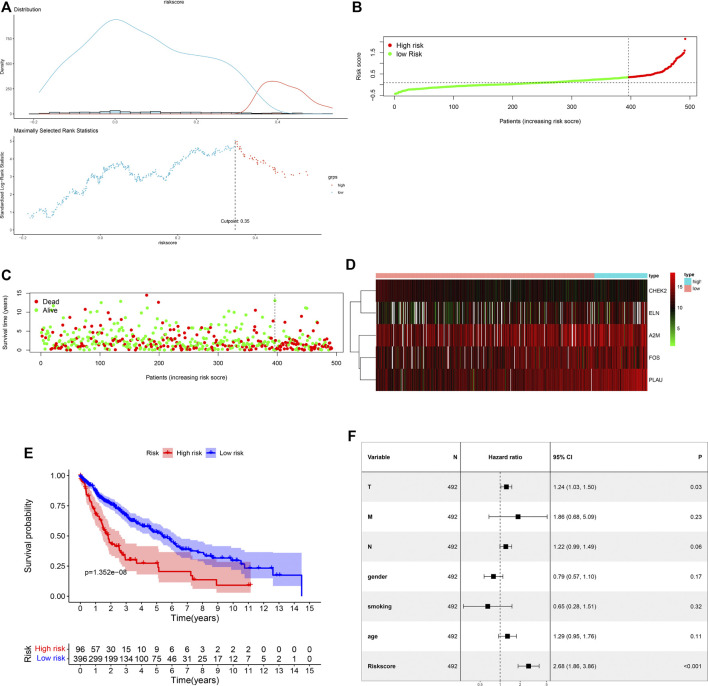
Assessment of prognostic value of the ARG signature model in the TCGA training cohort. **(A)** Determination of cut-off value of ARG risk scores by the maximally selected log-rank statistics. **(B)** The distribution of risk scores in the TCGA cohort. **(C)** Patient distribution in the high- and low-risk groups according to overall survival status. **(D)** The heatmap showing expression profiles of the five ARGs. **(E)** Kaplan-Meier curves for the overall survival of patients in the high- and low-risk groups. **(F)** Multivariate Cox regression analysis of the ARG signature and other clinicopathological factors.

In the training cohort, the cut-off value of risk scores was defined as 0.35 by means of the maximally selected log-rank statistics (Figure 3A), according to which we divided cases into low-risk and high-risk groups. Figure 3B shows the distribution of risk scores. As shown in Figure 3C, there were significantly fewer deaths due to LUSC in the low-risk patients than high-risk patients. We plotted a heatmap to show different expression levels of these five ARGs between the two risk groups (Figure 3D). Additionally, patients in the low-risk group had a significantly better OS than patients in the high-risk group (*p =* 1.352e-08) (Figure 3E). After integrating age, gender, smoking history, and T, N, and M classification, the risk score was still significantly associated with OS (HR = 2.68, 95%CI = 1.86–3.86, *p <* 0.001) (Figure 3F).

### Prognostic Value of the ARG Signature in the Validation Cohort

**FIGURE 4:**
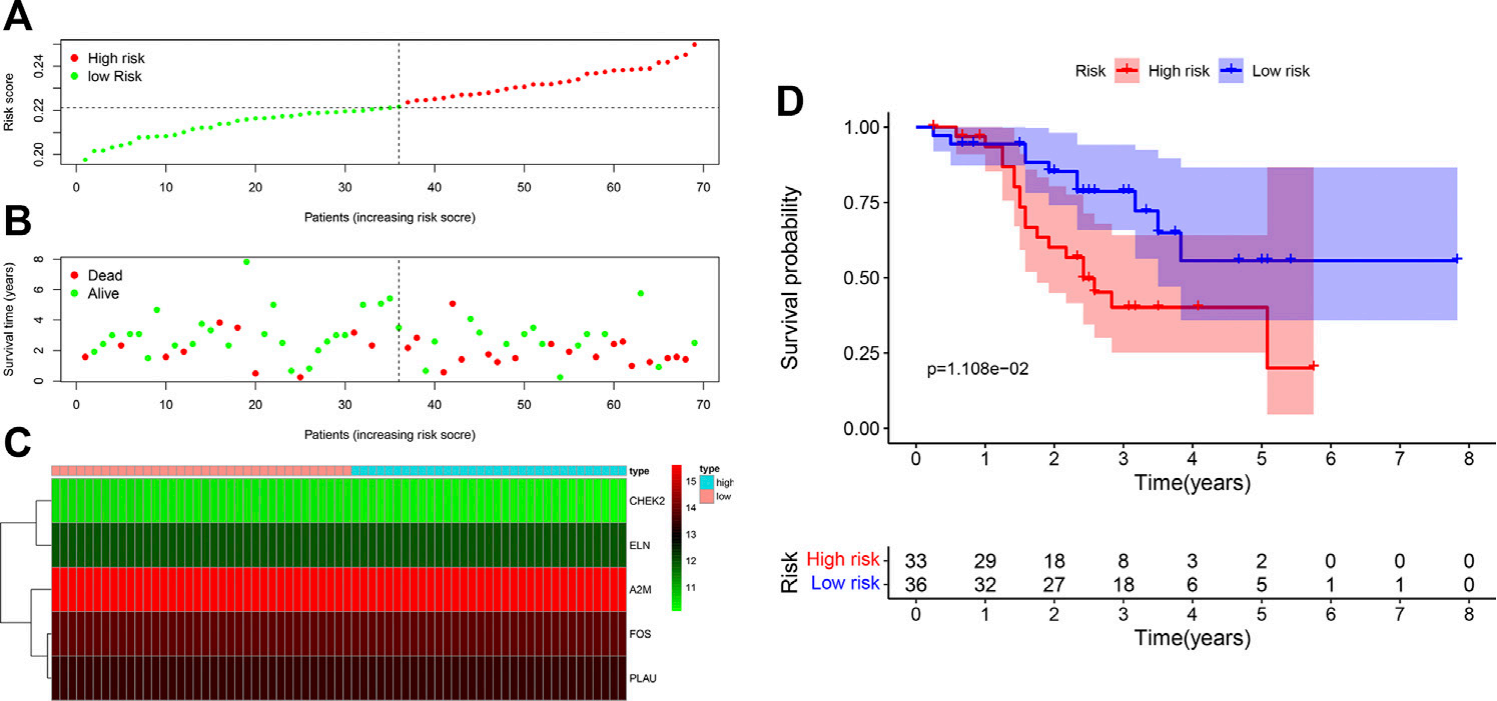
Assessment of prognostic value of the ARG signature model in the GSE20685 validation cohort. **(A)** The distribution of risk scores in the GSE20685 cohort. **(B)** Patient distribution in the high- and low-risk groups according to overall survival status. **(C)** The heatmap showing expression profiles of the five ARGs. **(D)** Kaplan-Meier curves for the overall survival of patients in the high- and low-risk groups.

In our validation cohort, 69 patients with LUSC were divided into high-risk (N = 33) and low-risk (N = 36) groups on the basis of the cut-off value of the risk score from the maximally selected log-rank statistics. The distribution of risk score is presented in Figure 4A. Similar with the training cohort, more deaths in the high-risk group were significantly found compared with the low-risk group (Figure 4B). As shown in Figure 4C, expression profiles of the five ARGs between low-risk and high-risk groups were plotted in the heatmap. The Kaplan-Meier curves revealed that patients in the low-risk group had an apparently longer OS than patients in the high-risk group (*p* = 1.108e-02) (Figure 4D). Further, the multivariate Cox model revealed the ARG risk score as an independent indicator for OS after controlling other clinical factors (HR = 9.51e+18, 95%CI = 752.28–1.20e+35, *p* = 0.02) (Table 2).

**TABLE 2:** Multivariate Analysis of GEO validation.

Factors	Multivariate analysis	
	HR (95%CI)	*p* Value
Gender		
Female		References
Male	1.68 (0.25–11.36)	0.59
Age (year)		
≤65		References
>65	1.01 (0.96–1.05)	0.81
Smoking history	1.77 (0.44–7.08)	0.42
T stage	1.86 (1.02–3.41)	0.04
N stage	1.50 (0.92–2.44)	0.10
M stage	—	—
Risk score	9.51e+18 (752.28–1.20e+35)	0.02

### Gene Set Enrichment Analysis for Important Pathways

**FIGURE 5:**
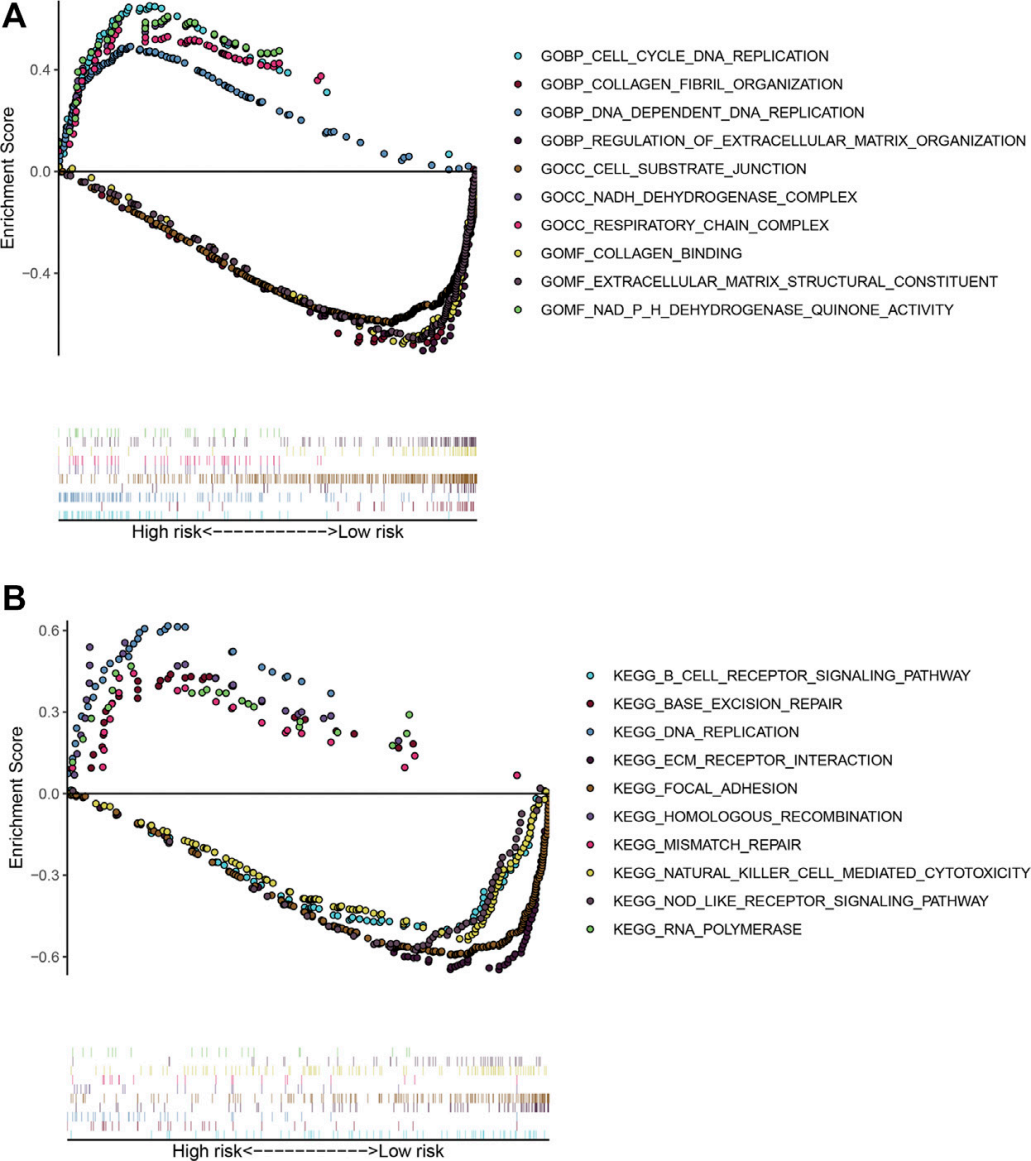
Gene set enrichment analysis between the low- and high-risk subgroups. **(A)** Enriched GO terms between high- and low-risk groups. **(B)** Enriched KEGG pathways between high- and low-risk groups.

To explore the potential functional mechanisms associated with the prognosis-related ARGs in patients with LUSC, we performed GSEA using GO and KEGG pathway enrichment analysis in the low-risk and high-risk groups in the TCGA training dataset. In patients with high-risk, genes were primarily enriched in collagen fibril organization, regulation of extracellular matrix organization, cell substrate junction, collagen binding, extracellular matrix structural constituent, ECM receptor interaction, focal adhesion, nod-like receptor signaling pathway, natural killer cell-mediated cytotoxicity, and B-cell receptor signaling pathway. In patients with low-risk, genes were primarily enriched in cell cycle DNA replication, DNA-dependent DNA replication, NADH dehydrogenase complex, respiratory chain complex, NADPH dehydrogenase quinone activity, base excision repair, DNA replication, homologous recombination, mismatch repair, and RNA polymerase (Figures 5A,B).

### Tumor Immunity Landscape and TMB in LUSC

**FIGURE 6:**
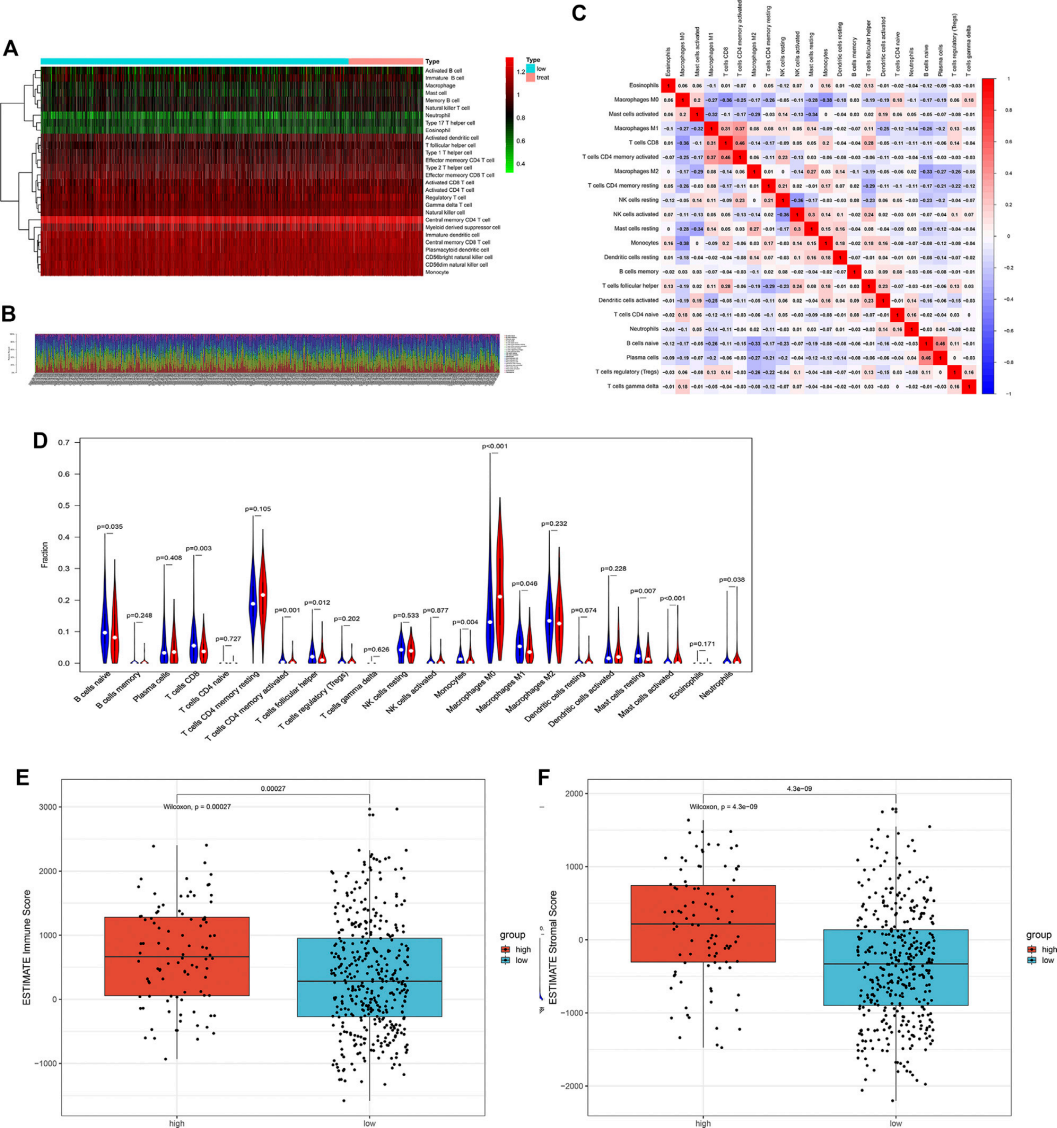
The landscape of immune cell infiltration between the high- and low-risk groups in the TCGA training cohort. **(A)** Heatmap of the 28 tumor-infiltrating cell proportions in ssGSEA. **(B)** Barplot of 22 immune cell infiltrations in CIBERSORT. **(C)** Correlation matrix of the association between the expression level of the five ARGs and tumor-infiltrating immune cell infiltrations. **(D)** Violin plot showing differences of infiltrating immune cell types between the low- and the high-risk groups. **(E)** Expression of the immune score between the low- and the high-risk groups. **(F)** Expression of the stromal score between the low- and the high-risk groups.

**FIGURE 7:**
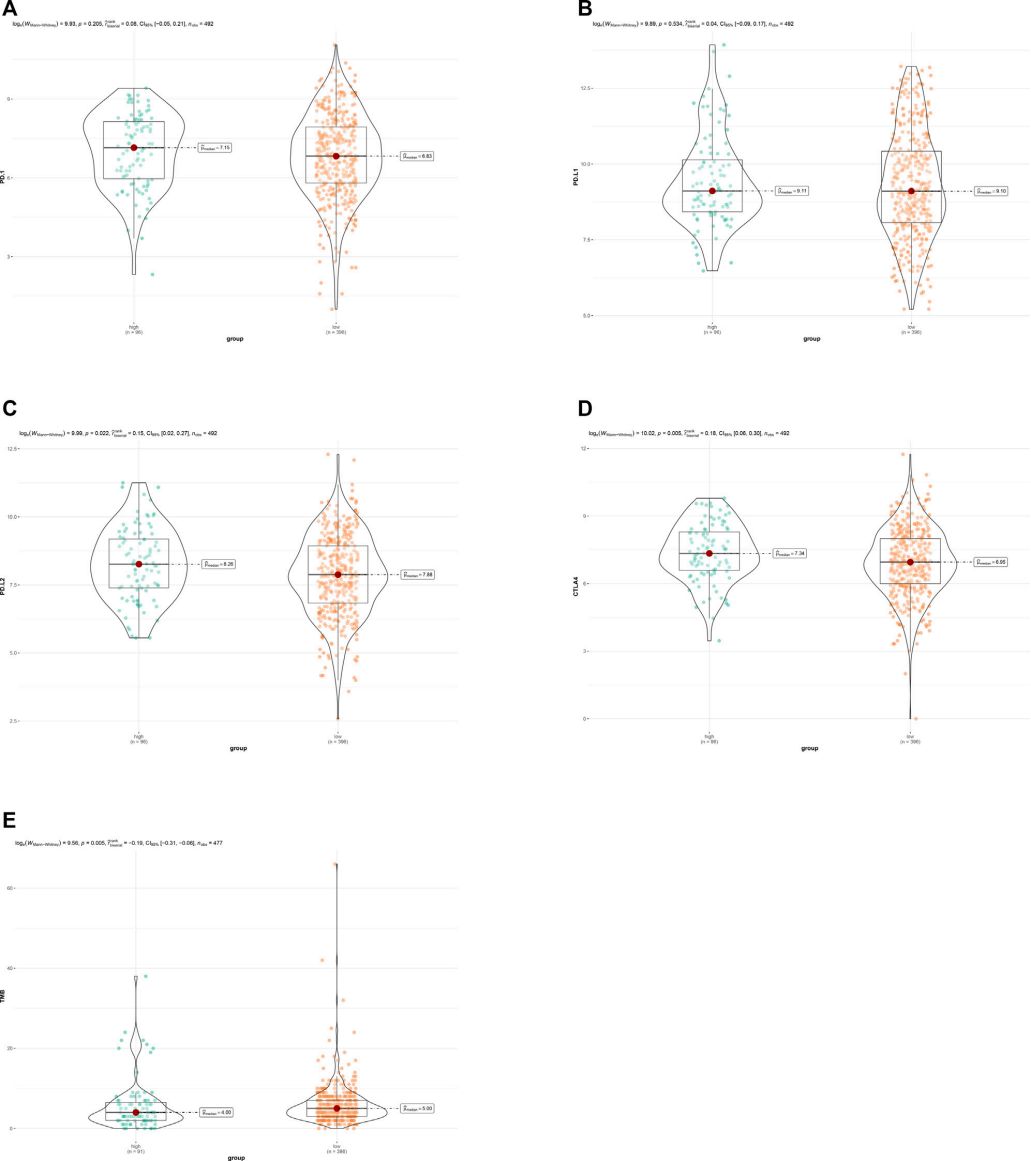
**(A)** Expression of PD-1 between the low- and the high-risk groups. **(B)** Expression of PD-L1 between the low- and the high-risk groups. **(C)** Expression of PD-L2 between the low- and the high-risk groups. **(D)** Expression of CTL4 between the low- and the high-risk groups. **(E)** TMB between the low- and the high-risk groups.

To explore the association between ARG risk scores and anti-tumor immunity, ssGSEA and the CIBERSORT algorithm were used to evaluate the immunity landscape between low-risk and high-risk groups in the TCGA dataset. The heatmap showed the results of ssGSEA in 28 immune cells, which demonstrated that these two risk groups had significantly different proportions of different immune cell infiltration (Figure 6A). Similar results were also seen in the CIBERSORT algorithm analysis of 22 immune cells (Figure 6B). Correlations among the 22 immune cell types are plotted in Figure 6C. As shown in Figure 6D, infiltrating proportions of naive B cells, CD8^+^ T cells, activated CD4^+^ memory T cells, follicular helper T cells, M1 macrophages, resting mast cells, and monocytes were apparently higher in low-risk patients while infiltrating proportions of M0 macrophages, activated mast cells, and neutrophils were significantly higher in high-risk patients. A significantly higher calculated immune score and stromal score with the characteristic of “hot tumor” were found in the high-risk group (Figures 6E,F). Compared with LUSC patients in the low-risk group, patients in the high-risk group tended to have a higher expression of PD-1 (Figure 7A), but no significant difference in the expression level of PD-L1 was found (Figure 7B). Patients with high-risk had an apparently higher expression level of PD-L2 and CTLA4 (Figures 7C,D), but patients with low-risk had a significantly higher TMB (Figure 7E).

### Nomogram Based on ARG Signature for LUSC

**FIGURE 8:**
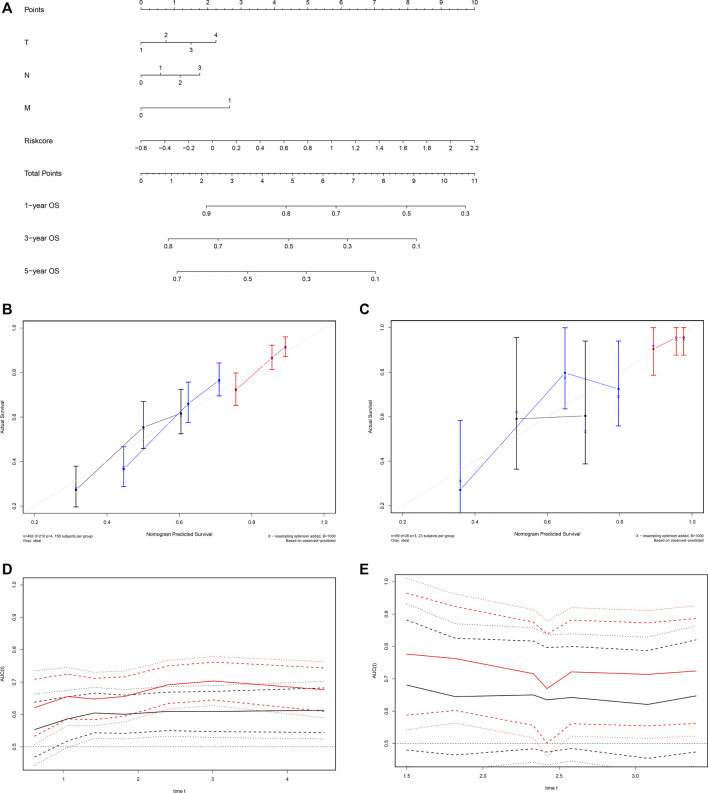
Development of a nomogram based on the ARG signature for predicting overall survival of patients with LUSC. **(A)** The nomogram plot integrating ARG risk score and T-, N-, and M-classification in the TCGA training cohort. **(B)** The calibration plot for the probability of 1-, 3-, and 5 years OS in the TCGA training cohort; 1-year: red; 3 years: blue; 5 years: black. **(C)** The calibration plot for the probability of 1-, 3-, and 5 years OS in the GSE73403 validation cohort; 1 year: red; 3 years: blue; 5 years: black. **(D)** Time-dependent ROC curves comparing the prognostic accuracy of the risk score combining ARGs and the TNM system in the training cohort; risk score + TNM: red; TNM only: black. **(E)** Time-dependent ROC curves comparing the prognostic accuracy of the risk score combining ARGs and the TNM system in the validation cohort; risk score + TNM: red; TNM only: black.

We established a visualized predictive nomogram model incorporating the ARG risk scores and T-, N-, and M-classification to predict individual OS probability at 1-, 3-, and 5 years using the data of the training cohort (Figure 8A). Bootstrap validation was performed in this nomogram. The C-index of the TCGA training cohort was 0.628 (95% CI: 0.586–0.671) and the C-index of the GSE73403 validation cohort was 0.648 (95% CI: 0.535–0.762), which suggested its good performance in predicting OS for LUSC patients. Calibration curves were drawn in both the TCGA cohort (Figure 8B) and the GSE73403 validation cohort (Figure 8C), which showed the good consistency between the actual survival and the nomogram-predicted survival at 1-, 3-, and 5-years. The time-dependent ROC curves showed that ARG risk scores combined with the TNM system has better capability in predicting OS for both training and validation cohorts (Figures 8D,E).

### Validation of the Expression Levels of Five ARGs in LUSC and Paracancerous Normal Tissues

**FIGURE 9:**
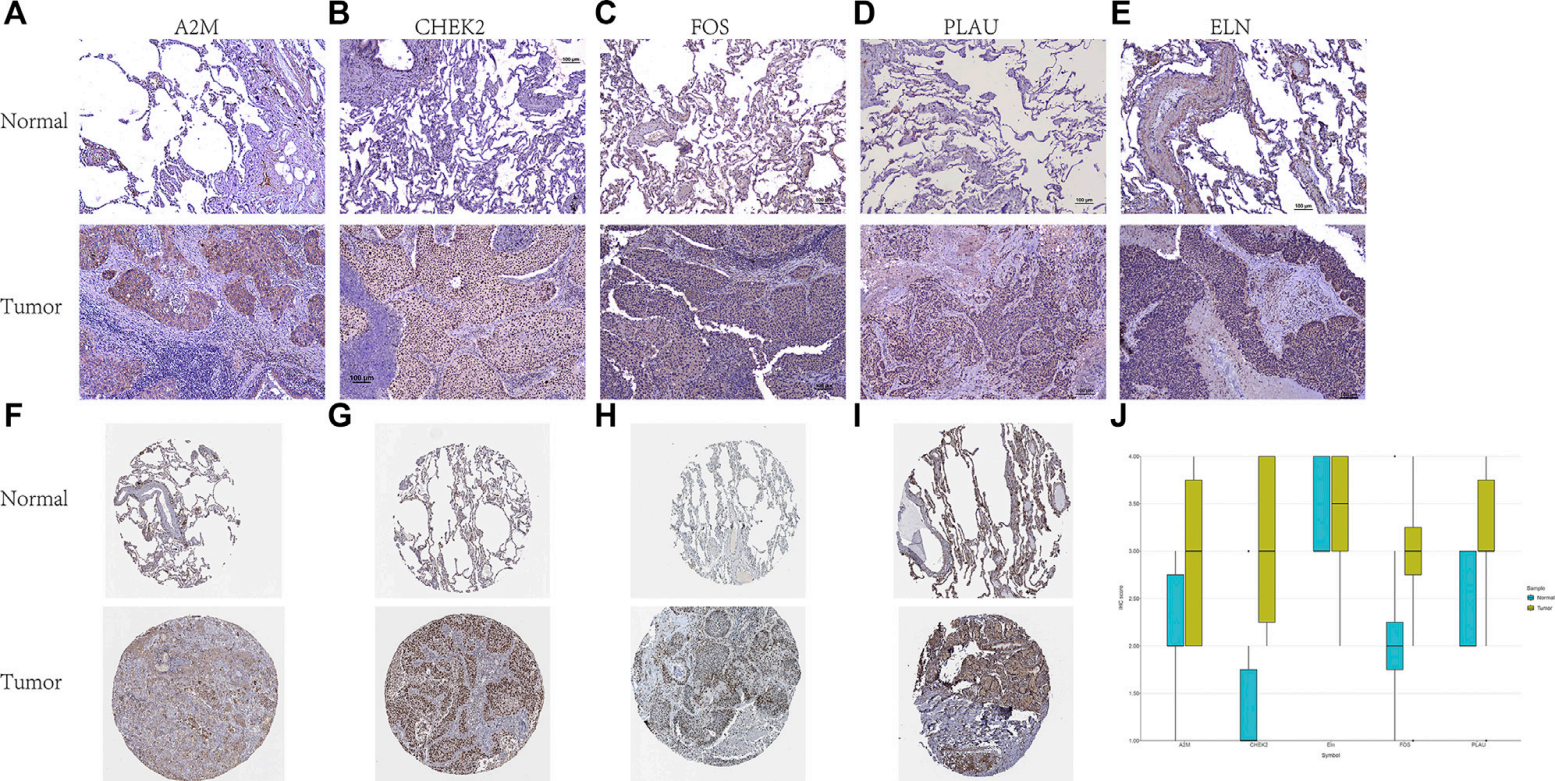
The representative images of IHC staining of five ARGs from SYSUCC. **(A)** A2M; **(B)** CHEK2; **(C)** FOS; **(D)** PLAU; and **(E)** ELN. The representative images of IHC staining of four ARGs from Human Protein Atlas. **(F)** A2M; **(G)** CHEK2; **(H)** FOS; and **(I)** PLAU. **(J)** The IHC score of 5 ARGs.

To verify the reliability of the results, tumor and adjacent non-tumorous tissue specimens from 10 LUSC samples were collected to test the expression levels of the five ARGs by IHC. The representative images of IHC staining of A2M, CHEK2, FOS, PLAU, and ELN are shown in Figures 9A–E. We also obtained the IHC staining images of A2M, CHEK2, FOS, and PLAU from the Human Protein Atlas database (Figures 9F–I). We found that the IHC scores of the five ARGs in tumor tissues were higher than those in the normal lung tissues (Figure 9J).

## Discussion

In the current study, we investigated the relationship between the expression levels of ARGs and survival of LUSC patients, and established a novel prognostic ARG signature consisting of five ARGs, i.e., A2M, CHEK2, FOS, ELN, and PLAU. In the TCGA training dataset, multivariate Cox analysis further revealed the independent prognostic value of the ARG signature. Then a predictive nomogram integrating this ARG signature and the TNM staging system was developed for predicting individual prognosis, and we validated its prognostic accuracy in the GSE73403 validation cohort. Furthermore, we explored the relation between the ARG risk signature and immune cell infiltration in patients with LUSC.

With the accessibility of getting free data from the public TCGA and GEO databases, more and more studies focus on the relation between RNA-seq data of specific gene sets and individual outcomes (Wu et al., 2019; Qu et al., 2020; Zhu et al., 2020). These studies were limited to autophagy, immune infiltration, and so on, and they lacked clinical extension. Besides, studies about the prognostic role of ARGs in LUSC are rare. Biologically speaking, along with the decline of function, aging is spontaneous and inevitable (Shavlakadze et al., 2019). Pathophysiologically speaking, metabolic disorders, declining immune response, and malnutrition occur during the aging process, and are a risk factor of many chronic illnesses, such as cancer (Smetana et al., 2016; Lee and Schmitt, 2019). Aging may also promote occurrence, development, and metastasis of tumors (Johnson et al., 2013; Mosteiro et al., 2016; Galluzzi et al., 2018; Calcinotto et al., 2019; Lee and Schmitt, 2019). A previous study revealed that ARGs were associated with the prognosis of lung carcinoma (Xu and Chen, 2021). Understanding the association between the ARGs and LUSC is also necessary and meaningful.

The ARG risk score formula of this study indicated that a high level of plasminogen activator urokinase (PLAU) gene expression was mostly unfavorable for individualized survival. Belonging to the plasminogen activator family, PLAU is a protease which is involved in cell migration and adhesion by activating several signaling pathways. A previous study reported that PLAU was associated with immune cell infiltration in LUSC (Zhang et al., 2020). Elastin (ELN), a fibrous protein, provides characteristic elasticity properties in several tissues (Salesse et al., 2018) and aberrant expression of ELN was a risk factor for lung fibrotic diseases (Li et al., 2020). But the underlying mechanisms and function of ELN have not been exhaustively studied in LUSC. The expression product of the FOS gene is c-FOS protein, which can dimerize with JUN family proteins to regulate downstream gene expression and participate in proliferation, invasion, metastasis, angiogenesis, and apoptosis of tumors (Angel and Karin, 1991; Hennigan et al., 1994; Milde-Langosch, 2005). The overexpression of FOS was also related to poor survival outcomes of LUSC (Volm et al., 1993). The protein encoded by A2M is alpha-2-macroglobulin, which may promote tumor progression in mice (Kurz et al., 2018). A previous study revealed that A2M was a hub gene significantly associated with the occurrence and development of LUSC (Zhang et al., 2019). Checkpoint kinase two is a pluripotent kinase encoded by the CHEK2 gene, which is associated with DNA repair, cell cycle regulation, and causes apoptosis when DNA damage occurs (Wang et al., 2014). CHEK2 gene mutation is also a pathogenic mutation in lung cancer and increases susceptibility to lung cancer (Liu et al., 2020). IHC staining discovered that the four ARGs, A2M, ELN, FOS, and PLAU, were overexpressed in LUSC, which is consistent with our ARG signature. However, IHC staining revealed that CHEK2 was overexpressed in tumor tissues. The IHC images in the Human Protein Atlas also demonstrate that CHEK2 was overexpressed in tumor tissues. Therefore, the possible mechanism of CHEK2 in LUSC needs further study.

Cellular senescence can lead to cancer-related immune responses, and the immune cellular infiltration in the tumor microenvironment contributes to the response of immunotherapy (Zhao et al., 2015). However, the relationship between immune cellular infiltration and aging in LUSC is poorly known. In this study, we discovered that patients in the low-risk group had an apparent increase in CD8^+^ T cell, activated CD4^+^ memory T cell, follicular helper T cell, and M1 macrophage infiltration. Results of the CIBERSORT algorithm show a favorable immune status in the low-risk group, which is associated with prolonged survival (Sebestyen et al., 2020; Zhang et al., 2020). However, the results of the ESTIMATE algorithm showed that patients with high-risk had a higher immune score and stromal score and a higher expression level of PD-L2 and CTLA4, which indicated that patients with a high-risk score had a more complex tumor immune microenvironment and more immune cell infiltration, although the immune cell infiltration in high-risk tumors did not show an anti-tumor effect. These results suggested that the high-risk group had greater potential to benefit from immunotherapy. In addition, patients in the low-risk group had a higher TMB, which related to a poor prognosis in NSCLC (Devarakonda et al., 2018). The Checkmate 026 trial determined that NSCLC patients with TMB ≥10/MB could benefit from immunotherapy (Carbone, et al., 2017), given both risk groups had a TMB less than 10/MB, TMB in this study could not be used as a predictor of immunotherapy.

There remain some limitations in our study. Firstly, this ARG prognostic model was only established by bioinformatic analysis from the public TCGA and GEO databases, results of this study need further validation from prospective, multicenter, or experimental data. Secondly, this study preliminarily explored the potential relationship between the ARG risk signature and anti-tumor immunity cell infiltration, studies are needed to reveal the underlying mechanisms by experimental data. Thirdly, although the ARG signature and TNM staging system were integrated in our prognostic nomogram, we cannot identify the contribution of each ARG in this signature.

In conclusion, the ARG risk score was associated with OS in patients with LUSC, we developed and validated a predictive nomogram for LUSC including the ARG risk signature and TNM staging system for predicting individual clinical prognosis. Moreover, we identified that patients with a high ARG risk score may have higher sensitivity to immunotherapy.

## Data Availability

Publicly available datasets were analyzed in this study. This data can be found here: TCGA databases (https://tcga-data.nci.nih.gov/tcga/), GEO databases (https://www.ncbi.nlm.nih.gov/geo/) and Human Protein Atlas (https://www.proteinatlas.org/).
