# Supplementary material for: An Aging-Related Gene Signature-Based Model for Risk Stratification and Prognosis Prediction in Lung Squamous Carcinoma
Source: Front Cell Dev Biol. 2022 Mar 1;10:770550. doi: 10.3389/fcell.2022.770550 (PMC8921527; doi:10.3389/fcell.2022.770550)
Supplement: Supplementary file 2 [file Table2.DOCX]

| Factors | Univariate Analysis | |
| --- | --- | --- |
|  | HR (95%CI) | P value |
| A2M | 1.000003669 (1.000000271-1.000007068) | 0.03433225 |
| CHEK2 | 0.999832205 (0.999668579-0.999995858) | 0.044476673 |
| ELN | 1.000022636 (1.000000384-1.000044889) | 0.046178027 |
| FOS | 1.000009417 (1.000002768-1.000016067) | 0.005502897 |
| PLAU | 1.000019577 (1.000011058-1.000028095) | 6.65E-06 |
